# The association of academic burnout with self-efficacy and quality of learning experience among Iranian students

**DOI:** 10.1186/2193-1801-2-677

**Published:** 2013-12-18

**Authors:** Morteza Charkhabi, Mohsen Azizi Abarghuei, Davood Hayati

**Affiliations:** Department of Psychology, University of Verona, Lungadige Porta Vittoria, Verona 17 - 37129 Italy; Department of Psychology, Allameh Tabatabaei University, Tehran, Iran; Department of Psychology, Shahid Chamran University, Ahvaz, Iran

**Keywords:** Academic burnout, Self-efficacy, Quality of learning experience

## Abstract

The present study examines the relationship between academic burnout and quality of learning experience and self-efficacy among undergraduate students. The sample consisted of 233 undergraduate students (106 men and 127 women) who were selected by stratified random sampling method. The participants completed the Quality of Learning Experience Scale, Academic Burnout scale, and General Self-Efficacy scale. This study is particularly interesting in the context of Iran, known for its equality-striving and high-quality educational system. Iranian youth, compared with youth in many other countries, have a lower level of well-being. The antecedents of academic burnout are divided into two categories: internal and external variables. In most studies regarding to the issue, one category is used to predict the dependent variable. However, in this study we utilized both ones; self-efficacy was considered as internal and quality of learning experience was used as an external predictor. Correlation coefficients indicated that all relationships between academic burnout and its components with self-efficacy were statistically significant. Furthermore, academic burnout and all of its components had significant correlations with quality of learning experience. Also, the relationship between resources with emotional exhaustion and professor-student relationship with academic inefficacy were not significant. On the basis of the results, through our research, we will expand academic burnout literature by focusing on its external and internal antecedents. In addition, we conclude with theoretical and practical implications and propose a clear horizon for future researches.

## Introduction

Several factors may affect the academic performance of students. Some of these factors improve academic performance and the others have detrimental effects on this academic construction. One of the most recently studied factors which affect students’ academic performance is academic burnout. Traditionally, academic burnout is defined as a three dimensional syndrome which includes emotional exhaustion, depersonalization, and reduced personal accomplishment (Maslach and Jackson, 1981 ). Burnout among college students refers to tiredness result from academic demands (emotional exhaustion), having a pessimistic sense and lack of interest toward academic tasks (cynicism), and feeling of incompetency as a student (inefficacy) (Shaufeli et al. 2002a, cited by Zhang et al. 2007 ). Evidence indicate that people with academic burnout may experience signs such as lack of interest toward academic issues, inability for attending in academic classes continuously, disaffiliation in class activities, sense of meaninglessness in academic activities, and incapability in acquisition of academic issues (Yang and Farn, 2005 ).

Neuman ( 1990 ) argues that academic burnout in students is one of the most significant fields of study in universities for several reasons. First, academic burnout can be the main reason of different behaviors in students such as academic performance; second, it can influence the relationship between students and their faculty in universities; and ultimately, academic burnout can affect students’ enthusiasm toward education. Therefore, it is definitely expected to reduce academic burnout by improving the academic achievement and learning motivation in students (Yang, 2004 ).

One of the known variables which can affect academic burnout is the quality of learning experience. This term was firstly defined by Neuman ( 1990 ). The quality of learning experience implies students’ perceptions toward direct and indirect inputs that they have received from college or university. For prevention and intervention, there have been conducted several studies and research to diagnose the antecedents and consequences of academic burnout which include environmental and individual aspects (Langelaan et al. 2006; Maslach et al. 2001; Shaufeli and Bakker, 2004; cited by Zhang et al. 2007 ).

In the past decades, investigators have addressed self-efficacy as one of the effective individual factors in explaining burnout phenomenon (Shaufeli et al. 1993; cited by Yang and Farn, 2005 ). Self-efficacy is defined as the belief that a person can do something successfully (Woolfolk, 2004 ). Some of previous experiments have confirmed the relationship between self-efficacy and burnout in describing the relationship between burnout and physiological manners (Yang and Farn, 2005 ;Hobfoll and Freedy, 1993 ). Recently conducted investigations indicate that self-efficacy is a good predictor of academic burnout among students (Brouwers and Tomic, 2000 ). Moreover, later researches about the relationship between self-efficacy and burnout concluded that self-efficacy beliefs are negatively associated with depersonalization and emotional exhaustion and are positively correlated with decreased personal accomplishment in students (Evers et al. 2002 ). These findings displayed that a higher score in self-efficacy indices is along with the decreased burnout symptoms in persons. Other evidence showed that emotional exhaustion has a closed relationship with academic stress (Murberg and Bru, 2004 ). Also, Pessimistic attitude and cynicism toward academic issues can expectedly decline learning motivation (Deci et al. 1991 ) and the feeling of failure can be related to decreased self-esteem (Saunders et al. 2004 ). Previous research in the field of industrial and organizational psychology have shown that burnout has several negative consequences such as absenteeism, turnover, poor health, and higher level of depression (Toppinen-Tanner et al. 2005 ). Duran et al. ( 2006 ) investigated the predictors of academic burnout and academic engagement and concluded that stress and overall self-efficacy are related to academic burnout and academic engagement. By the way, by controlling these two variables, they identified that emotional intelligence can also serve as a predictor of academic burnout. In addition, Shwarzer and Hallum ( 2008 ) in the two separated studies (first one among 1203 teachers and the second one among 458 teachers) about job pressure and burnout in Germany and Syria found out that teacher’s self-efficacy is correlated with their job pressure and burnoiut. Furthermore, current researches have shown that faculty climate and received positive motivation from professors have a negative significant relationship with academic burnout (Salmela-Aro et al. 2008 ;Kiura et al. 2008 ). In fact, the most important novelty of the research is to investigate internal and external predictors of academic burnout simultaneously.

Considering the past and current researches in the above, this study wants to investigate the relationship between individual self-efficacy and external quality of learning experience with academic burnout. Thus, the hypotheses will be:

1. There is a negative significant relationship between quality of learning experience and academic burnout.
  1.1. There is a negative significant relationship between quality of learning experience and academic inefficacy.
  1.2. There is a negative significant relationship between quality of learning experience and cynicism.
  1.3. There is a negative significant relationship between quality of learning experience and emotional exhaustion.
2. There is a negative significant relationship between self-efficacy and academic burnout.
  2.1. There is a negative significant relationship between self-efficacy and academic inefficacy.
  2.2. There is a negative significant relationship between self-efficacy and cynicism.
  2.3. There is a negative significant relationship between self-efficacy and emotional exhaustion.

## Method

### Sample

In this study, the sample consisted of *233* undergraduate students (*106 men and 127 women, M = 21*) of Allamme Tabatabaei University of Iran who were selected by stratified random sampling method form different faculties and different fields.

### Data collection

Data were collected through questionnaire method (survey) where self-reported questionnaires were administered. Closed ended questions were used in gathering information. Participants had adequate time to respond to the asked questions. Accurate and reliable data were collected through questionnaire method and hence it is deemed very efficient. Participants studied through this method are given adequate time and freedom to answer the questions and hence accurate information is collected. Using from the closed ended questions helps in restricting participants to some questions and gathering wide range of information respectively. The selected persons were informed about the needed instructions for filling out self-reported surveys. At the end of this step, we could get a collection of various contributions.

### Measures

#### Quality of learning experience scale

This scale is developed by Neuman ( 1990 ) which evaluates four different aspects of quality of learning experience including: resources (such as, research facilities, the quality of library and computer); content (such as, the quality of academic guidance and the value of presented academic issues); learning flexibility (such as, the existence of independent learning opportunity, ability for choosing different courses and free discussion in class); and finally, the quality of formal and informal relationship between professors and students in university. Neuman ( 1993 ) found the reliabilities of these four aspects (resources, content, flexibility and quality) are 0.71, 0.74, 0.86, and 0.91 respectively. Also, Neuman ( 1993 ) reported the validity of the scale in a range of 0.35 to 0.67.

#### Academic burnout scale

Breso et al. ( 2007 ) designed this scale to measure the burnout level in students. This scale contains 15 items which evaluate the dimensions of emotional exhaustion (5 items), cynicism (4 items), and academic efficacy (6 items). Participants must indicate the amount of agreement to each item, which were scored on Likert response scale from 1 (totally disagree) to 4 (totally agree). The reliability of these three dimensions (emotional exhaustion, cynicism, and academic efficacy) was 0.70, 0.82, and 0.75 respectively.

#### General self-efficacy scale

Sherer, et al. ( 1982 ) developed a scale which is a 17 item scale. This scale measures the generalized expectations that having successful experience in the past can indicate future success. Participants answer the questions based on Likert response scale from 1 (totally disagree) to 5 (totally agree). The Chronbach alpha of the scale is 0.86. Also, the validity of the scale as a measurement of general self-efficacy has been supported by several studies (Sherer et al. 1982Sherer and Adams 1983 ).

## Results

In descriptive part of statistical results, we measured the frequency and percentage of participants which were 106 men (45.5%) and 127 women (54.5%). Being student made them excused of any other demographic characteristics such as tenure and so on. Therefore, the other characteristics did not take into our study account. Table 1 displays the results of correlation between self-efficacy and academic burnout and its components. As this is obvious, all findings are significant in p < 0.01 level.

**Table 1:** **Correlation coefficients of self-efficacy with academic burnout and its components**

Academic burnout and its components	Emotional exhaustion		Inefficacy		Cynicism		Academic burnout	
**Self-efficacy**	- 0.34*	.001	- 0.22*	.001	- 0.35*	.001	- 0.37*	.001

*P < 0.05.

Thus, based on Table 1, it can be concluded that all relationships between self-efficacy with academic burnout and its components were significant. Also, Table 2 shows the results of the relationships between the quality of learning experience and its sub-scales with academic burnout and its components.

**Table 2:** **Correlation coefficients between quality of learning experience and its sub-scales with academic burnout and its components**

	Emotional exhaustion	Cynicism	Academic inefficacy	Academic burnout
**Quality of learning experience**	-0.18*	-0.29***	-0.21***	-0.28***
**Reference**	-0.12	-0.14*	-0.15**	-0.17**
**content**	-0.17**	-0.23***	-0.26***	-0.27***
**Flexibility**	-0.13*	-0.26***	-0.16**	-0.23***
**Professor-student relationship**	-0.13*	-0.24***	-0.8	-0.18**

*P < 0.05, **P < 0.01, ***P < 0.001.

According to Table 2, academic burnout and all of its components had significant relationship with quality of learning experience. It is worth to mention that the relationships of resources with emotional exhaustion and professor-student relationship with academic inefficacy were not significantly.

## Discussion

The aim of this study was to investigate the relationship between quality of learning experience and self-efficacy with academic burnout among undergraduate students. Generally, the antecedents of academic burnout are divided into two categories: internal and external variables. In most studies regarding to this issue, one category is used to predict the dependent variable. However, in this study we utilized both ones; self-efficacy was considered as internal and quality of learning experience was used as an external predictor.

The results showed that all considered relationships were significant. Some prior researchers have confirmed the relationship between self-efficacy and burnout to describe the association between burnout and physiological states (Cherniss, 1993 ; Hallsten, 1993 ; Hobfoll, and Freedy, 1993 ). They have shown that people who are without skill feeling (e.g. feeling of efficacy) lose the capability of adaptation. Bandura ( 1977 ) applied his social-cognitive theory to explain the concept of efficacy. Efficacy beliefs have gained increasing attention in educational researches, especially regarding to academic motivation and self-determination (Pintrich and Schunk, 1995 ). Hence, people with high level of self-efficacy when are facing with academic problems, they are not giving up and try to find useful solutions for their problems. Furthermore, they experience lower stress because they are using of planning. It is obvious that stress is one of the factors which can lead to burnout.

About the relationship between resource and academic burnout, it should be mentioned that lack of resource for doing the academic tasks can lead to shaping the context of burnout. In the other word, undergraduate students are under pressure to do different duties because they need sufficient facilities and resources. When the academic tasks are a lot and the students do not have enough resources, they will experience higher levels of stress, and their ability and motivation for doing these tasks will be declined. Among critical needed resources, library and computer sites should be under more attention by deans and principals. To some extent that they sufficiently are not available; the higher amount of stress will be experienced. This chronic stress can result in burnout (Halbesleben and Buckley, 2004 ). Therefore, a combination of high academic demands and low resources can produce higher academic burnout (Bakker et al. 2003 ).

Students mainly have intense relationships with their professors during the academic course; however, the quality of this communication can impact their emotions and feelings profoundly. Based on some studies (Fraser, 1994, 1997 ), there are two behavioral categories which are related to professors. The first one is named organizer behavior. Determining regular schedule, goal setting and assigning ordered tasks to students can influence academic achievement. The other one is supportive behavior. The communication in which professors are sensitive toward their students’ feelings and self-concept, and they sympathize with them that can play a meaningful role in reduction of academic burnout (Naami, 2009 ).

At the end, it should be noted that studying the association of burnout with other related variables (e.g. stress, social support) is suggested to get a wider attention. Then, professors and teachers are asked to increase the self-efficacy level among their students to predict or control burnout. It can be gained by presenting some tasks which can enforce personal achievements. In addition, verbal reinforcement is the other method to augment self-efficacy. Howsoever, considering the significant role of external factors in academic burnout (e.g. quality of learning experience), principals and deans are suggested to prepare sufficient equipment and resources such as equipped and updated libraries and computer sites. Being flexible and creating a friendly climate by the teachers and professors in class are the two other pleas which can be suggested. Finally, it is needed to consider that these students would be the future makers.

## References

[CR1] Bakker AB, Demerout E, Taris TW, Schaufeli WB, Schreurs PJG (2003). A multi group analysis of the job-demands-resource model in four home care organizations. Int J Stress Manag.

[CR2] Bandura A (1977). Self-efficacy: toward a unifying theory of behavioral change. Psychol Rev.

[CR3] Breso E, Salanova M, Schoufeli B (2007). In search of the third dimension of burnout. Appl Psychol.

[CR4] Brouwers A, Tomic W (2000). A longitudinal study of teacher burnout and perceived self-efficacy in classroom management. Teach Teach Educ.

[CR5] Cherniss C, Shaufeli WB, Maslach C, Marek T (1993). Role of professional self-efficacy in the etiology and amelioration of burnout. Professional burnout: recent development in theory and research.

[CR6] Cotton K (1997). Educating for citizenship.

[CR7] Deci EL, Vallerand RJ, Pelletier LG, Ryan RM (1991). Motivation and education: the self-determination perspective. Educ Psychol.

[CR8] Duran A, Extremera N, Rey L, Fernandez-Berrocal P, Montalban F (2006). Predicting academic burnout and in educational settings: assessing the incremental validity of perceived emotional intelligence beyond perceived stress and general self-efficacy. Psicotherma.

[CR9] Evers WJG, Brouwers A, Tomic W (2002). Burnout and self-efficacy: a study on teachers’ beliefs when implementing an innovative educational system in the Netherlands. Br J Educ Psychol.

[CR10] Fraser BJ (1994). Research on classroom and school climate, handbook of research on science teaching and learning.

[CR11] Halbesleben JR, Buckley MR (2004). Burnout in organization life. J Manag.

[CR12] Hallsten L, Schaufeli W, Maslach C, Marek T (1993). Burning out: a framework. Professional burnout. Recent developments in theory and research.

[CR13] Hobfoll SE, Freedy J, Schanfeli WB, Maslach C, Marek T (1993). Conservation of resource: a general stress theory applied to burnout. Professional burnout: recent developments in theory and research.

[CR14] Kiura N, Aunola K, Numi J (2008). Peer group influence and selection in adolescents school burnout. Merrill Palmer Q.

[CR15] Maslach C, Jackson SE (1981). The measurement of experienced burnout. J Occup Behav.

[CR16] Murberg TA, Bru E (2004). School-related stress and psychosomatic symptoms among Norwegian adolescents. Sch Psychol Int.

[CR17] Naami A (2009). The relationship between quality of learning experience with academic burnout among MA students of Shahid Chamran University of Ahvaz. Psychol Stud.

[CR18] Neuman Y (1990). Determinants and consequences of students’ burnout in universities. J High Educ.

[CR19] Neuman Y (1993). Quality of learning experience and students college outcomes. Int J Educ Manag.

[CR20] Pintrich PR, Schunk DH (1995). Motivation in education: theory, research, and application.

[CR21] Salmela-Aro K, Savolainen H, Holopainen L (2008). Depressive symptoms and school burnout during adolescence. J Youth Adolesc.

[CR22] Saunders J, Davis L, Williams T, Williams JH (2004). Gender differences in self-perceptions and academic outcomes. J Youth Adolesc.

[CR23] Sherer M, Adams CH (1983). Construct validation of self-efficacy scale. Psychol Rep.

[CR24] Sherer M, Maddux JE, Mercandante B, Prentice-Dunn S, Jacobs B, Rogers RW (1982). The self-efficacy scale: construction and validation. Psychol Rep.

[CR25] Shwarzer R, Hallum S (2008). Perceived teacher self-efficacy as a predictor of job stress and burnout: mediation analyses. Appl Psychol.

[CR26] Toppinen-Tanner S, Ojaarvi A, Vaananen A, Kalimo R, Jappinen P (2005). Burnout as a predictor of medically certified sick-leave absences and their diagnosed causes. Behav Med.

[CR27] Woolfolk A (2004). Educational psychology.

[CR28] Yang H (2004). Factors affecting student burnout and academic achievement in multiple enrollment programs in Taiwan’s technical-vocational colleges. Int J Educ Dev.

[CR29] Yang H, Farn CK (2005). An investigation of the factors MIS student burnout in technical- vocational college. Comput Hum Behav.

[CR30] Zhang Y, Gan Y, Cham H (2007). Perfectionism, academic burnout and engagement among Chinese college students: a structural equation modeling analysis. Personal Individ Differ.

